# A diverse and multi-modal gait dataset of indoor and outdoor walks acquired using multiple cameras and sensors

**DOI:** 10.1038/s41597-023-02161-8

**Published:** 2023-05-26

**Authors:** Luke K. Topham, Wasiq Khan, Dhiya Al-Jumeily, Atif Waraich, Abir J. Hussain

**Affiliations:** 1 Liverpool John Moores University, Liverpool, UK; 2 University of Sharjah, Sharjah, United Arab Emirates

**Keywords:** Research data, Technology

## Abstract

Gait datasets are often limited by a lack of diversity in terms of the participants, appearance, viewing angle, environments, annotations, and availability. We present a primary gait dataset comprising 1,560 annotated casual walks from 64 participants, in both indoor and outdoor real-world environments. We used two digital cameras and a wearable digital goniometer to capture visual as well as motion signal gait-data respectively. Traditional methods of gait identification are often affected by the viewing angle and appearance of the participant therefore, this dataset mainly considers the diversity in various aspects (e.g., participants’ attributes, background variations, and view angles). The dataset is captured from 8 viewing angles in 45° increments along-with alternative appearances for each participant, for example, via a change of clothing. The dataset provides 3,120 videos, containing approximately 748,800 image frames with detailed annotations including approximately 56,160,000 bodily keypoint annotations, identifying 75 keypoints per video frame, and approximately 1,026,480 motion data points captured from a digital goniometer for three limb segments (thigh, upper arm, and head).

## Background & Summary

The analysis of gait (*i.e*., the manner of a person’s walking), has proven to provide relevant clues regarding a person’s health^1^, and a means of re-identification^2,3^. Existing gait datasets are often limited by several aspects such as lack of participant diversity, low quality images or data, low quantity of data per participant, limited or inconsistent viewing angles, a lack of background variation, natural outdoor environments, and more^4^. Currently there is a lack of literature regarding the effect of variables such as age, gender, height, and mass on a person’s gait, likely due to the lack of a sufficiently diverse dataset. Furthermore, the datasets are often recorded in laboratory environments which do not reflect real-world environments^4^. Therefore, the lack of a gait dataset with significant diversity in these features is a major limitation to the related research domains.

To address these limitations, we aimed to collect a new gait dataset with a relatively large number of participants with significant diversity in terms of participants’ personal features such as age, gender, height, mass, and ethnicity, as well as other perspectives mainly including realistic environmental settings, varied viewing angles, multiple sensors etc, and made it publicly available^5^. For ethical and safety reasons, only participants over the age of 18 and without disabilities or injuries that may make walking uncomfortable or dangerous were considered for use in these experiments. We also aimed to record the data in realistic environments with a variety of backgrounds with the hope that any methods developed using the proposed dataset would more easily be deployed to real-world applications.

The dataset presents further opportunities in anthropometric gait analysis, in addition to applications of person re-identification (e.g., gait identification, face recognition, face de-identification, pose estimation, motion tracking, pedestrian walking, human body segmentation, gait rehabilitation etc.). We anticipate that this dataset will be of particular value to diverse applications of gait analysis and machine learning in various domains which will benefit from the quality and quantity of the data in addition to the diversity of the participants and recording environments (indoors/outdoors, and multiple viewing angles). In addition, the thorough labelling of the dataset, including frame by frame labelling of the video recordings, will assist many researchers in performing their work. Furthermore, person identification, including gait identification, poses additional requirements for datasets. For example, person identification has shown to be affected by factors such as viewing angle^6^, appearance (e.g., clothing and accessories)^7^, and environment. Therefore, for a dataset to be appropriate for the problem of gait identification, it must provide significant diversity in these areas to ensure that any identification models developed can perform accurately in real-world dynamic environments where the conditions cannot be controlled.

We collected a primary gait dataset from 64 participants which consists of 1,560 walking sequences in total (24 walking sequences of 8 metres per participant), recorded from two angles providing 3,120 videos. Each walk is recorded using two cameras (Kodak AZ422, 20MP) at different positions and angles, camera placements are altered to provide 8 different viewing angles per participant (See Fig. 3, 4). It may have been advantageous to secure a greater number of cameras to record the desired range of angles, however, availability of resources limited the authors to two cameras, hence the need to be creative in the design of the experiments to achieve the desired range of viewing angles. In total approximately 415 minutes of video and sensor recordings have been collected producing approximately 748,800 video frames in total (30 fps). In addition, a wearable digital goniometer sensor, MOTI^8^, was used to collect motion data regarding the leg, arm, and head movement during the walking (at 84 hertz), providing potential ground truth to potential computer-vision applications or data to aid the development of solutions for wearable sensors. Approximately, 1,026,480 samples were collected from the sensor in total.

In addition to aforementioned unique aspects we considered in our dataset, clothing and appearance has been shown to affect human body pose estimation and person re-identification^9^. A unique feature of the proposed dataset is that each participant, where possible, repeated several of the walking sequences while wearing alternative clothing. This will enable future work to evaluate the effect of personal appearance on applications such as person re-identification, or to build improved applications that minimise the effect.

### Related datasets

An overview of related datasets is provided in Table 1, additional detail is provided in Supplementary Table 1. Digital video recording offers a convenient approach for non-invasive gait analysis and gait identification. However, as digital cameras are relatively expensive and recording images of a person may been seen as an invasion of privacy by the participant, often datasets do not provide digital images. For example^10^, provides only IMU and electromyography (EMG) sensor data and^11^ provides only a processed gait silhouette image instead of the original digital images.

**Table 1:** Overview of Existing Related Gait Datasets Including a Survey of the Sensors, Environments, and Diversity.

Name	No. Subjects	No. Instances	Environment	Participant Diversity	Alternative Clothing/Appearance	Sensors	Labelling	Camera angles
HuGaDB^10^	18	679,073 frames	Indoor	Limited diversity, only 4 females	N/A	6 IMUs (upper legs, lower legs), feet, 2 EMGs.	Activities (e.g., walking, sitting)	N/A
Gait Silhouette Dataset^11^	30	300 walks 100,000 frames	Unknown	Not described	No	Digital camera.	None.	1
IST gait database^12^	21	252 walks	Outdoors	Not described	No	Digital Camera.	None	4
Human Gait Phase Dataset^13^	21	25,306 steps	Indoors	Limited age, height, and mass diversity. Diversity in gender split	No	Motion capture.	3D motion tracking. Ground reaction forces	1
Gait Recognition Image and Depth Dataset (GRIDDS)^14^	35	350 walks	Indoors	Limited	Yes	Depth camera, microphone.	Silhouette images	1
SOTON HiD^15^	~100	~800 walks ~1,200 steps	Indoors	Not described	No	Digital camera.	None	4
TUM Gait from Audio, Image and Depth (TUM-GAID)^16^	305	3,370 walks	Indoors	Limited (particularly in age and height, ethnicity not recorded)	For 32 participants only. Limited appearance diversity due to weather conditions. With and without a backpack	Depth camera, microphone.	None	2
Ours	64	1,560 walks, 3,120 videos, ~10,920 steps (per angle), 748,000 frames, >1 million sensor samples, >56 million annotated keypoints	Indoors and outdoors with background variations	Yes – gender, age, height, mass, and ethnicity	Yes. Alternative clothing for each participant	2 digital cameras, digital goniometer.	HPE (75 keypoints per image frame), anthropometry	8 (45° steps)

Furthermore, another common limitation is the number and diversity of the participants within the dataset. For example^10–14^, all contain less than 50 participants. Similarly, a low number of instances per participant are provided in^11,14^, thus limiting the ability to implement machine learning methods, especially deep learning. Moreover, diversity (e.g., environment, camera view, background variations, etc) within datasets is important, particularly when implementing machine learning models, it also provides the opportunity for anthropometric gait analysis, a current open problem. As far as the authors are aware, the datasets described in Table 1 do not provide significant diversity in terms of participants’ age, gender, height, mass, and ethnicity to perform anthropometric gait analysis. Diversity and realism in the recording environments may allow for real world applications to be developed and analysed, however, this can be a difficult task, particularly when using motion capture technology, for example, datasets such as^13,15^ are restricted to an indoor laboratory environment. Additionally, both datasets are also restricted to treadmill walking which may not produce natural walking styles for each participant. Finally, the availability of datasets particularly, video or image datasets is very limited due to ethical concerns, for example^16,17^, are not currently publicly available.

In contrast, our dataset provides 64 diverse participants with up to 24 instances each, recorded at multiple angles in real-world environments. Our dataset contains approximately 415 minutes of video, which provides approximately 748,800 frames. The dataset is publicly available and includes HPE labelling (approximately 748,800 frames, 56,160,000 labelled keypoints, and 1,026,480 labelled sensor data points in total), which is not available in any of the existing related datasets described in. Similarly, digital goniometer data is provided for three body limb segments, as far as the authors are aware, this sensor is not included in any current gait datasets.

## Methods

### Participants

A total of 64 participants were included in this dataset, only volunteers aged over 18 years, who declared that they did not have any medical conditions which affected their gait or posture were considered. Ethical approval was provided by the University Research Ethics Committee (UREC) at Liverpool John Moores University (LJMU) (Ref: 21/CMP/004), furthermore, all participants read the participant information sheet which explained what was involved in the recordings and how the data would be used, including a statement explaining that they would be identifiable in the video data. The participants then signed a consent form and completed a questionnaire prior to completing the recordings. Participant recruitment aimed to recruit a diverse range of participants in terms of gender, age, ethnicity, height, and mass. Participant diversity can be seen in Table 2, which presents the anthropometry information collected for each participant (also included in the dataset as participant information.xlsx). The data was collected from participants via a questionnaire, manual measurements (height and mass) were taken where participants were uncertain of their measurements. All participants are numbered (*i.e*., participant_1) to minimise personal information whilst still enabling person identification experiments. Participant 42 agreed to be included in the author’s dataset but not the public copy, therefore, the decision was made to maintain the record in the anthropometry records and dataset file structure to ensure all versions of the dataset are aligned.

**Table 2:** Anthropometry Information of the Participants (P_No) along with Ethnicity (Eth) where WB, B, BB, BBA, WD, WP, WI, WIT, AS, MR, AF, GR, AR, WE, BE, represents White British, Black, Black British, British-Bangladeshi, White Dutch, White Polish, White Irish, White Italian, Asian, Mixed-Race, African, Greek, Arab, White European, Black European, respectively.

P_No	Gender	Age	Height (cm)	Mass (kg)	Eth	P_No	Gender	Age	Height (cm)	Mass (kg)	Eth
1	F	27	153.0	60.0	WB	34	F	62	173.0	62.0	WB
2	F	32	159.0	63.0	WB	35	F	50	165.0	—	WB
3	M	30	175.0	65.0	AS	36	M	23	182.0	85.7	WB
4	F	57	153.0	95.0	WB	37	F	52	162.5	52.0	WB
5	M	29	170.0	80.7	WB	38	M	20	180.0	70.0	AR
6	M	27	177.0	108	WB	39	M	52	180.0	113.0	WB
7	F	53	155.0	63.5	WB	40	F	34	165.0	51.0	AS
8	M	27	169.0	80.0	WB	41	M	47	190.5	88.9	WB
9	F	42	169.0	76.2	WB	42	—	—	—	—	—
10	M	40	178.0	77.0	—	43	M	21	182.0	116.0	AR
11	M	37	180.0	70.0	AS	44	F	39	167.6	60.8	WB
12	M	37	171.0	67.0	MR	45	F	40	137.2	133.4	WB
13	F	32	165.0	75.0	MR	46	M	62	177.8	100.7	WB
14	M	21	185.0	95.0	AS	47	F	29	170.2	57.2	WB
15	M	19	181.0	90.0	AS	48	F	21	173.0	61.0	WD
16	M	32	180.0	86.0	AS	49	F	27	160.0	67.1	WB
17	M	55	172.7	90.0	AS	50	F	31	157.5	83.5	WI
18	M	21	175.3	85.0	AS	51	F	39	160.0	53.0	WIT
19	M	30	182.9	75.0	AS	52	F	26	152.4	74.9	WB
20	M	28	183.0	70.0	WB	53	F	27	157.5	99.8	WB
21	M	31	185.0	117.5	WB	54	M	47	177.0	72.0	MR
22	M	25	183.0	79.5	WB	55	F	23	157.0	56.0	BBA
23	M	65	165.0	82.5	WB	56	F	51	165.0	92.0	WB
24	F	42	152.0	133.0	WB	57	M	61	174.0	98.5	WB
25	M	25	188.0	77.0	WB	58	F	18	162.5	54.0	WB
26	F	24	162.5	57.0	WB	59	M	36	180.3	85.0	WB
27	M	25	175.0	67.0	AF	60	F	25	162.6	98.0	WB
28	M	40	172.0	95.3	BB	61	F	26	170.2	80.0	WB
29	M	22	172.0	63.0	WB	62	M	30	165.0	66.0	AR
30	M	20	173.0	74.0	WB	63	M	40	180.0	81.0	AF
31	F	45	160.0	69.9	WB	64	M	29	180.0	90.0	WP
32	M	44	167.0	79.0	B	65	M	26	192.0	110.0	MR
33	M	34	177.0	107.0	GR	**Summary**	**M: 37** **(57.81%)** **F: 27** **(42.19%)**	**35.14 (±12.44)** **Max: 65** **Min: 18**	**170.83 (±10.86)** **Max: 192** **Min: 137.16**	**81.51 (±20.18)** **Max: 133.36** **Min: 51**	**WE: 62.5%** **BE, AR, AS, AF: 37.5%**

**Fig. 1:**
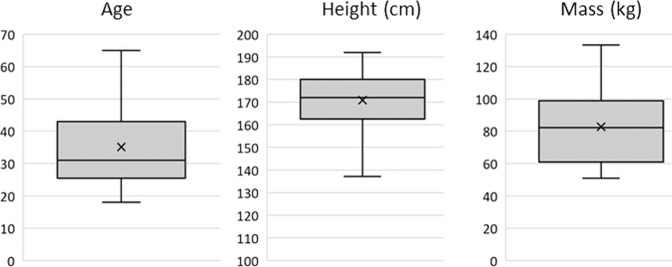
Box plots of Participants’ Age (years), Height (cm), and Mass (kg) Distributions.

**Fig. 2:**
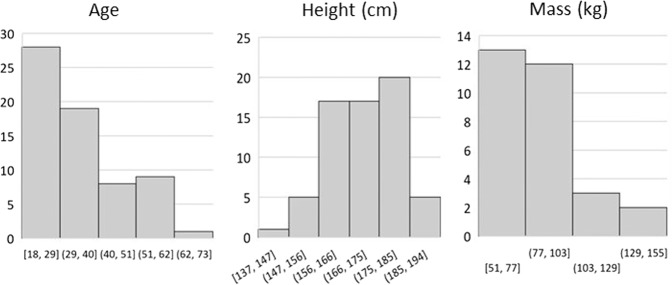
Histograms of Participants’ Age (years), Height (cm), and Mass (kg) Distributions.

Moreover, of the 64 participants in the dataset 38 (58.46%) are male and 27 (41.54%) were female. The age range of 18 to 65 covers most of the range of adult working ages in the UK. The average height (170.83 cm ± 10.86) and mass (81.51 kg ± 20.18) of the participants is close to that of the average European and the standard deviations suggest that there is ample dispersion of values in the dataset. Although most participants (62.5%) recorded their ethnicity as white European, this lower than the national average of England and Wales (80.5%^18^), suggesting that the dataset is more diverse than the nation in which it was recorded. The remaining 37.5% of participants were Black European, Arab, Asian, or African. Furthermore, Figs. 1, 2 display the distribution of the ages, heights, and masses of the participants in the dataset via box plots and histograms respectively, all of which suggest significant diversity. In additional to participant diversity, a variety of real-world backgrounds are provided in the dataset, including both indoor and outdoor settings. Outdoor recordings are provided for participants 14 to 19 inclusively, accounting for approximately 8% of all recordings, the remainder of which were recorded in indoor environments.

### Experimental tasks

Three groups (henceforth known as experiments) of 6 walking tasks were required of each participant (extended to 8 tasks with the repetition of tasks 1 and 2 where alternative clothing is available). Each experiment corresponds to a different camera layout, and each task in every experiment required the participants to walk for 8 metres. The participants were instructed to walk casually in their normal manner, in terms of their walking style and speed. To aid the participants, they were instructed to imagine that they were taking an un-rushed walk to a shop.

**Fig. 3:**
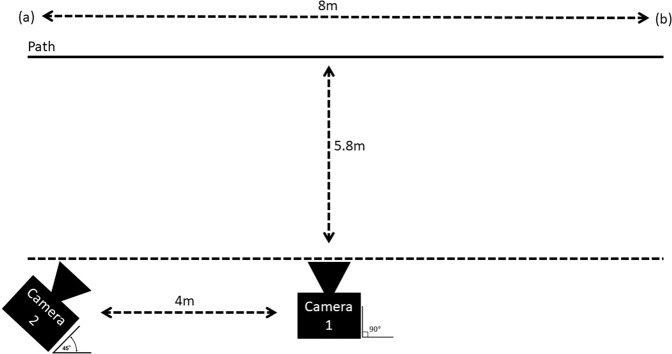
Camera Layout and walking paths for all tasks in Experiments 1 and 2 (Experiment 2 Reversed Image).
- Experiment 2: Camera layout and walking path as per Fig. 3 (mirrored).
  - Task 1: Sensor placement right leg, walk from a to b.
  - Task 2: Sensor placement right leg, walk from b to a.
  - Task 3: Sensor placement right arm, walk from a to b.
  - Task 4: Sensor placement right arm, walk from b to a.
  - Task 5: Sensor placement forehead, walk from a to b.
  - Task 6: Sensor placement forehead, walk from b to a.
- Experiment 3: Camera layout and walking path as per Fig. 4.
  - Task 1: Sensor placement right leg, walk from a to b.
  - Task 2: Sensor placement right leg, walk from b to a.
  - Task 3: Sensor placement right arm, walk from a to b.
  - Task 4: Sensor placement right arm, walk from b to a.
  - Task 5: Sensor placement forehead, walk from a to b.
  - Task 6: Sensor placement forehead, walk from b to a.

The complete experiment and tasks list is as follows:

- Experiment 1: Camera layout and walking path as per Fig. 3.
  - Task 1: Sensor placement right leg, walk from a to b.
  - Task 2: Sensor placement right leg, walk from b to a.
  - Task 3: Sensor placement right arm, walk from a to b.
  - Task 4: Sensor placement right arm, walk from b to a.
  - Task 5: Sensor placement forehead, walk from a to b.
  - Task 6: Sensor placement forehead, walk from b to a.

**Fig. 4:**
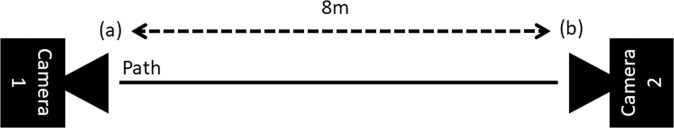
Camera Layout and walking paths for all tasks in Experiment 3.

### Video capture

Two cameras are used to record each walking task, which for the 3 experiments provide 12 different recording angles for each participant, as the participants alternate the walking direction with each task (*i.e*., they walk in one direction, and then walk from the opposite direction in the following task). The arrangement of the cameras and the variety of walking direction effectively provides a 360° view of each participants’ walking, in 45° increments.

All tasks for each experiment require the participants to walk along a measured 8 metre path, as displayed in Figs. 3, 4. In experiment 1 (see Fig. 3), one camera is placed perpendicular to the walking path at exactly halfway (4 metres) along the path, with a gap of 5.8 metres between the path and the camera. A second camera is placed opposite the starting position of the odd numbered tasks. The starting position for the participants alternates between each end of the walking path with each task. The alternating starting point provides two benefits, firstly, both sides of the participants body are recorded, secondly, camera 2 captures the participants walk from two different angles across each pair of recordings.

Similarly, experiment 2 may be considered as a reflected or reversed image of experiment 1 (see Fig. 3). To achieve this, the camera positions, and the walking path for experiment 1 as shown in Fig. 3 are simply swapped. Again, this achieves additional recording angles of each participant’s walk, in addition to a different background.

Furthermore, experiment 3 also includes an 8-metre walk; however, the cameras are place directly along the walking path, as shown in Fig. 4. This provides a frontal and rear view of each participants walking. Again, as with the previous experiments, the starting points of each task alternate and therefore two different backgrounds are captured in this experiment.

**Fig. 5:**
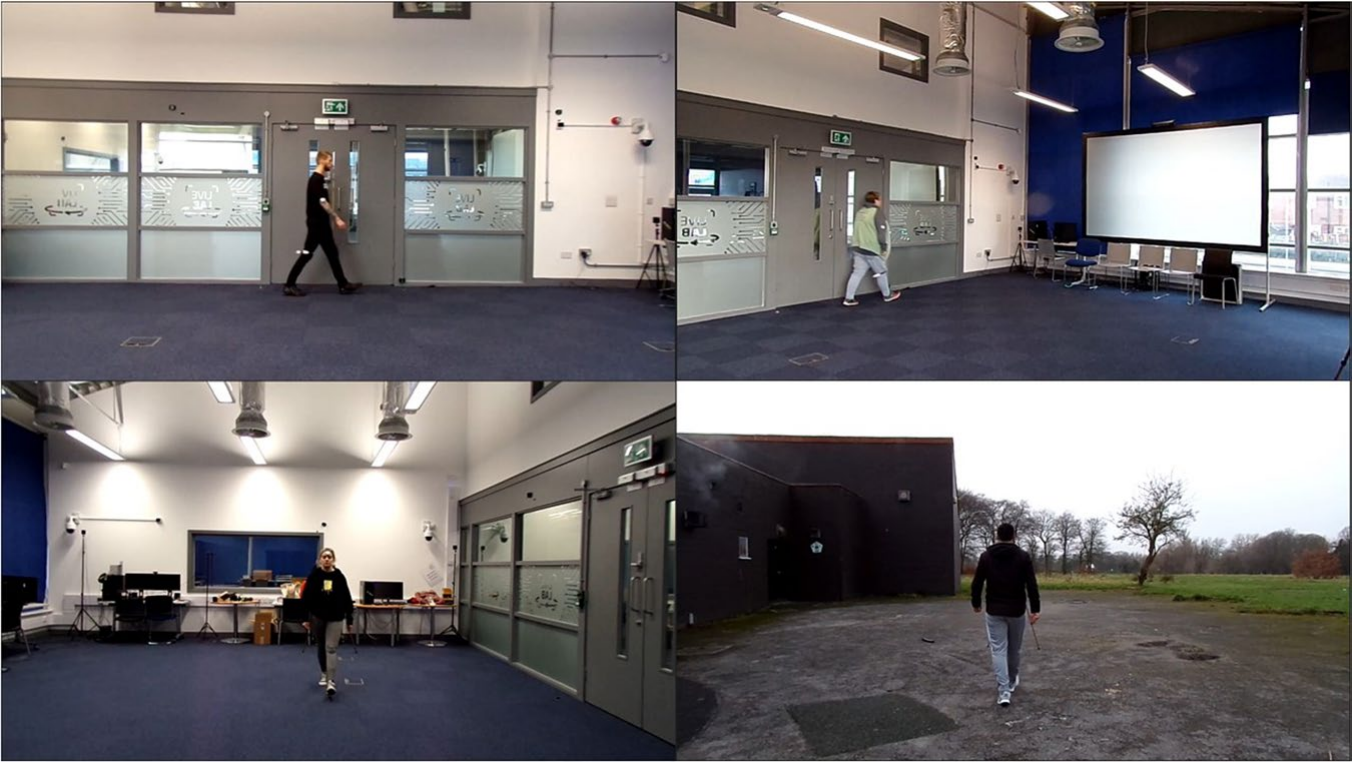
Still Image Samples of Camera Angles and Backgrounds from Recorded Data (Image shared with participant’s consent).

Still image examples of the variety of camera angles (4 examples from the available 8 angles), and backgrounds acquired during the recording process are shown in Fig. 5. Efforts were made to ensure that the locations and backgrounds used provided real-world environments for the data capture.

### Goniometer sensor

In each experiment, the participants also wear a digital goniometer sensor. A goniometer is a device often used by physiotherapists to measure the Range of Motion (ROM) of bodily joints or limb segments^19^. In these experiments, a digital goniometer developed by MOTI^8^ is used, which is built using an Inertial Measurement Unit (IMU). The IMU includes an accelerometer to measure acceleration, a gyroscope to measure angular velocity, and a magnetometer to measure the magnetic field of the earth^20^. The tri-axial sensor data is then harnessed to calculate the ROM of the sensor, and by extension, the limb segment responsible for the movement, similar to the methods described in^21,22^. The sensor is attached to the participant using an adjustable strap which enables it to be quickly attached and detached. The position of the sensor is alternated with every second task in each experiment to record the thigh, upper arm, and head limb segments. These limb segments were chosen due to their relative height difference in relation to the participants. This will allow the authors’ and other researchers in the area of gait identification to prototype methods of gait identification which may be applied to the problem of occlusion (for example, computer vision-based approaches to gait identification where the subject is walking behind a wall or other obstacle).

**Fig. 6:**
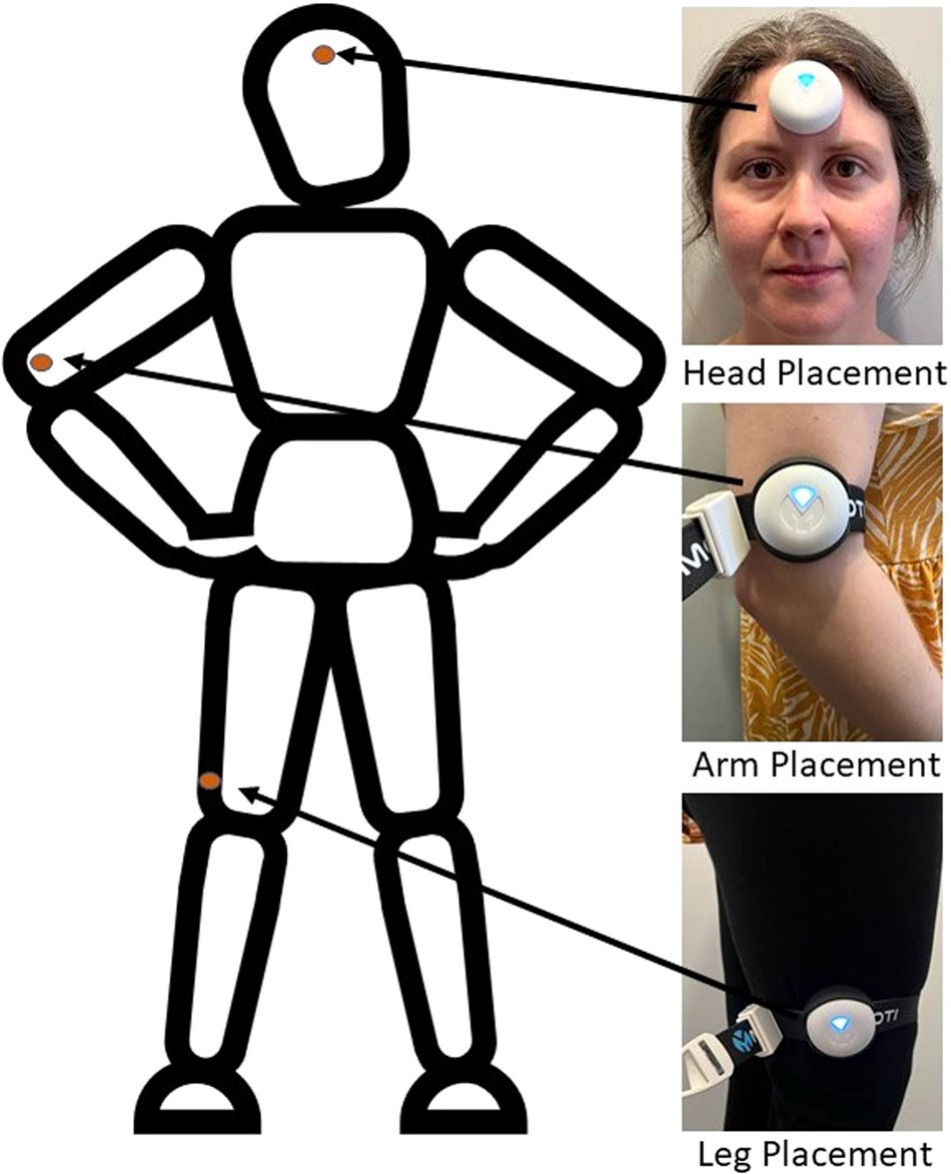
Goniometer Sensor Placements (right leg, right arm, and forehead) (Image shared with participant’s consent).

In tasks 1 and 2, the sensor is attached to the participant’s right leg, just above the knee. This position allows the device to record the movement of the leg at the thigh. In tasks 3 and 4, the sensor is attached to the participant’s right arm, just above the elbow. This position allows the device to record the movement of the arm at the upper arm. Finally, in tasks 5 and 6 the device is attached to the participant’s forehead using a double-sided sticker, this allows the device to measure the movement of the head from the neck. Figure 6 displays the various sensor placements. All data is recorded via an application on a mobile phone in .csv format, these files are then copied to a desktop computer. A total of 1,560 motion data files were collected providing approximately 1,026,480 time-stamped samples, in which approximately 12,480 gait cycles are present. The .csv file generated by the goniometer for each task contains a variety of data from each of the IMU sensors as described in Table 3.

**Table 3:** Description of Digital Goniometer Data Columns and Corresponding Units.

Column	Unit	Description
Timestamp	Seconds (s)	Experiment runtime
q0	Quaternion	Imaginary number satisfying Hamilton’s equation^24^.
q1	Quaternion	Imaginary number satisfying Hamilton’s equation^24^.
q2	Quaternion	Imaginary number satisfying Hamilton’s equation^24^.
q3	Quaternion	Imaginary number satisfying Hamilton’s equation^24^.
ROM	Degrees (°)	Limb segment angular motion.
Roll	Degrees (°)	Rotation around the longitudinal axis.
Pitch	Degrees (°)	Rotation around the transverse axis.
Yaw	Degrees (°)	Rotation around the vertical axis.
accX	Metres per second squared (m/s^2^)	Acceleration in the X axis provided by the accelerometer.
accY	Metres per second squared (m/s^2^)	Acceleration in the Y axis provided by the accelerometer.
accZ	Metres per second squared (m/s^2^)	Acceleration in the Z axis provided by the accelerometer.
gyrX	Degrees per second (°/s)	Rotation measurement around the X axis as a scalar value.
gyrY	Degrees per second (°/s)	Rotation measurement around the Y axis as a scalar value.
gyrZ	Degrees per second (°/s)	Rotation measurement around the Z axis as a scalar value.
magX	Miilitesla (mT)	Measurement of the magnetic field for the X axis.
magY	Miilitesla (mT)	Measurement of the magnetic field for the Y axis.
magZ	Miilitesla (mT)	Measurement of the magnetic field for the Z axis.

### Data collection

Approximately 415 minutes of video recordings, containing approximately 748,800 video frames (excluding the extracted skeleton videos) were collected across both cameras (i.e., ~207.5 minutes per camera). This corresponds to approximately 1,560 walking tasks which in turn contain approximately 12,480 complete gait cycles. Moreover, while recording at the digital goniometers default frequency of 84 hertz approximately 1,026,480 time-stamped samples are collected.

### Data processing

All recorded data was transferred from the relevant devices to a desktop computer for post-processing. The goniometer sensor recording is manually started and stopped at the beginning and ending of each task and therefore does not require any post-recording processing.

As the video is recorded continuously during each experiment, some processing of the video data is required. Firstly, the video must be cropped so that each recorded walking task is separated into individual video files. This is achieved using video editing software (Windows Photo application), each task is manually cropped so that each video starts at the first sign of movement after the audible start command and ends at either the stopping of motion or where the participant disappears from the camera view, whichever occurs first. The video and sensor data are manually synchronised for each recording. The sensor is manually started and stopped when the command to the participants is verbally given. The videos are then carefully cropped so that the duration is equal to that of the sensor data. This is achieved by aligning the start and stop points using the motion clues available in both the video (visible movement) and sensor data (detected movement). The videos provided in the dataset have all been cropped using the aforementioned video cropping process.

**Fig. 7:**
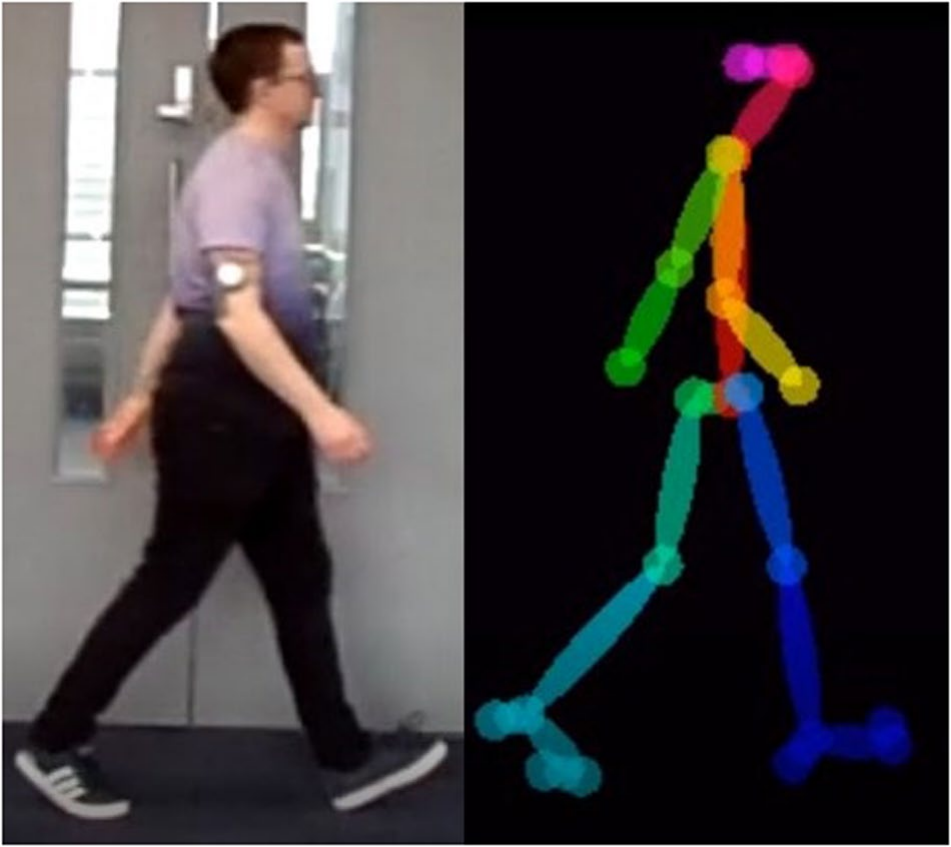
Comparison of Original Frame (left) and OpenPose^23^ Skeleton Frame (right) (Image shared with participant’s consent).

Furthermore, to aid future work, additional labelling is provided in the form of Human Pose Estimation (HPE). Using the pre-trained OpenPose 2D HPE^23^ system 75 anatomical keypoints are labelled on the participant’s body for each frame of each video. This processing provides estimated skeletal movement tracking which may aid areas of research including gait analysis and gait identification. All authors manually performed a visual check of the labelling accuracy before adding the data to the final version of the dataset. From the 1,560 collected walks, approximately 748,800 frames have been labelled, which provide a total of approximately 56,160,000 labelled keypoints. The labelling is provided in two ways. Firstly, a folder is created for each video file to hold a JSON file for each frame in the video, this file contains the location of the keypoints detected in the frame. Additionally, a video is generated from the JSON files so that each of the original videos also has a corresponding video (.avi format) containing only the extracted “skeleton” generated from the anatomical keypoints in the JSON files. An example of an extracted and visualised skeleton is provided in Fig. 7 alongside the original video frame (cropped for convenience here).

## Data Records

**Fig. 8:**
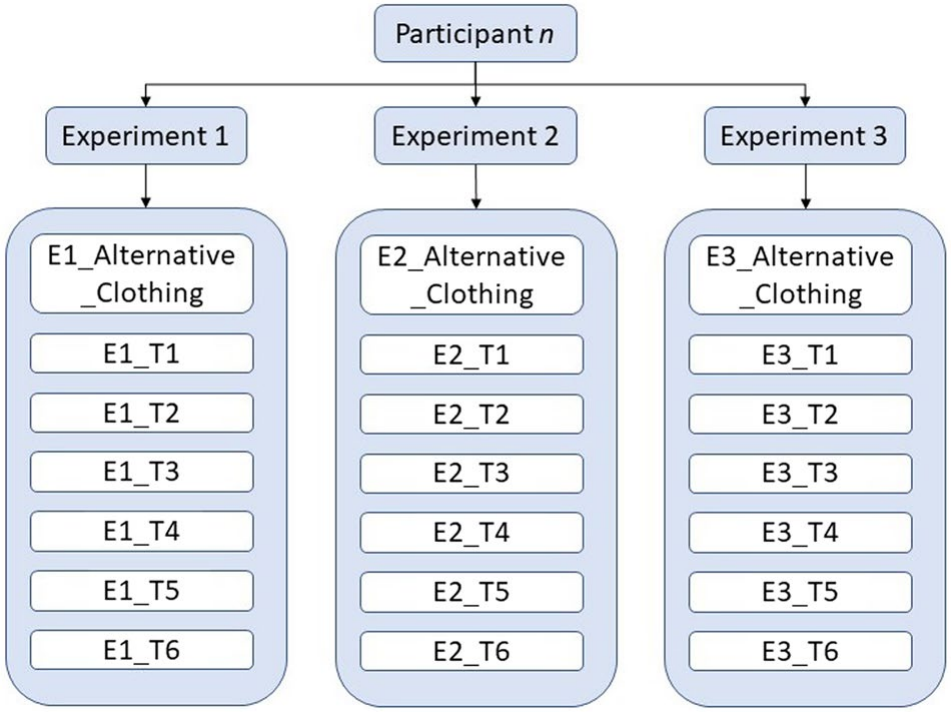
The Datasets Folder Layout Per Participant Containing 3 Experiment (e.g., Experiment_1) Folders Each and 7 Task folders within them (e.g., E1_T1 represents Experiment 1 Task 1).

We provide the described dataset via the LJMU data repository (https://opendata.ljmu.ac.uk/id/eprint/133/)^5^. The repository contains a folder which provides a copy of the anthropometry information of the participants (participant Information.xlsx) in addition to one folder per participant. Each of the participant’s folder contain the entirety of the recorded data for that participant across all three experiments, as illustrated in Fig. 8. There is one subfolder for each of the three experiments, and inside them are six folders for each of the walking tasks within the experiment, plus one folder for the experiments where the participant wore alternative clothing (and repeated tasks 1 and 2). To make this clear to the user, the repeated task files are appended with “_ALT” to specify that alternative clothing has been worn for these repeated tasks. Finally, inside each of the task folders (e.g., E1_T1) the data for the task can be found, which includes two video files of the recorded walk in .mp4 format (*i.e*., two different angles of the same walking task), the corresponding extracted skeleton videos in .avi format, two folders containing the corresponding skeleton keypoints (one folder per video, one JSON file per frame), and a .csv file containing the digital goniometer data.

A simple file naming convention is used through the dataset to ensure each file is easily understood. A folder is assigned to each participant, labelled Participant_x, where x is the sequential number assigned to the participant. Inside of each participant folder are three experiment folders, labelled Experiment_1, Experiment_2, and Experiment_3, where an experiment (e.g., Experiment 1) is a group of individual recorded walking tasks. Inside the individual experiment folders (e.g., Experiment 1) each of the seven task sub-folders are labelled to include the experiment number followed by the tasks numbers, for example, the task folder E1_T1 corresponds to experiment 1, task 1. Moreover, inside the task folders, the files also contain the participant number, followed by the experiment number and task number, for example, the digital goniometer file P1_E1T1.csv corresponds to participant 1, experiment 1, task 1. Furthermore, the video files also include an appended camera angle. Here, _45 refers to a 45° recording angle, _90 refers to a 90° recording angle, _front represents a 0° recording angle (i.e., the participant is walking directly towards the camera), and _rear represents a 180° recording angle (i.e., the participant is walking away from the camera). For example, P1_E1T1_90 refers to the video recording of participant 1, experiment 1, task 1, recorded at a 90° angle (see Figs. 3, 4 for an illustration of the camera layouts for each experiment). Finally, “_ALT” is appended to video files within the alternative clothing folder to signify that the participant has repeated the experiment with alternative clothing worn in these records, as compared to those worn in the original task recordings.

Furthermore, the columns recorded by the digital goniometer device are described in Table 3. The columns include a timestamp, quaternions (q0, q1, q2, q3). The calculated measurement of the limb segment ROM is provided by the ROM column. Euler angles describing the movement of the sensor are provided by Roll, Pitch, Yaw. The acceleration of the sensor for the x, y, and z axis, as measure by the accelerometer, are provided in accX, accY, and accZ. The rotation of the sensor for the x, y, and z axis, as measured by the gyroscope, are provided in gyrX, gyrY, and gyrZ. Finally, the magnetometer sensor readings for the x, y, and z axis are provided in magX, magY, and magZ.

## Technical Validation

### Sensor placement and function

The sensors were placed on the participants according to the manufacturer’s instructions^8^, as shown in Fig. 6. An adjustable strap was used to attach the sensor to the participants ensuring that the sensor was firmly attached regardless of the clothing type worn by the participant. Each participant was requested to confirm that the placement and attachment of the sensor did not impede normal movement before each recording. Furthermore, before commencing each recording, the sensor signal was manually verified during a practice walk to ensure proper function. Manual inspections continued throughout the experiments to ensure proper function was maintained.

### Camera placement

Consistent camera placement (see Figs. 3, 4) was ensured via the manual measurement of the camera placement as described in the methods section. The distance between cameras, the walking path, position of the cameras, height of the camera, and angle of the cameras were all manually measured prior to each recording session.

### Quality assurance

All authors reviewed the collected dataset to ensure quality. For example, the collected dataset was split among each of the authors. Each author assessed their assigned portion of dataset for quality, including sensor quality, image quality and video processing quality (e.g., video cropping, data synchronisation, keypoint annotation). Furthermore, the authors were responsible for ensuring that only the experiment participants were captured in the video recording and that no bystanders were captured, an important ethical issue. Similarly, each author ensured that no unnecessary identifiable information was included in the videos, for example, house numbers, street names, and car registrations.

## Usage Notes

A folder is provided for each participant containing subfolders for each experiment and further subfolders for each of the walking tasks within those experiments. Each task folder contains two video recordings in .mp4 format, the corresponding skeleton videos in .avi format, a folder containing.JSON files for the skeletons in each frame in each of the videos, and finally a .CSV file containing the sensor recordings (the alternative clothing folder contains double, as it contains the repetition of both tasks 1 and 2). We do not provide code to extract data; however, this is a relatively trivial problem that can be implemented using standard tools such as pandas.

The data provides healthy gait data in real-world environments from a diverse range of participants. Potential applications or studies include, but are not limited to, person identification, or anthropometry focused gait analysis.

## Supplementary information

Supplementary Table 1. Overview of Existing Related Gait Datasets Including a Survey of the Sensors, Environments, and Diversity.

Supplementary Table 2. Anthropometry Information of the Participants.

## Data Availability

No custom code has been written for this dataset.

## References

[1] Khokhlova M, Migniot C, Morozov A, Sushkova O, Dipanda A (2019). Normal and pathological gait classification LSTM model. Artif. Intell. Med..

[2] Topham, L., Khan, W., Al-Jumeily, D., Waraich, A. & Hussain, A. J. Gait Identification using Hip Joint Movement and Deep Machine Learning. in *International Conference on Intelligent Computing* 220–233, 10.1007/978-3-031-13832-4_19 (Springer, 2022).

[3] Topham L, Khan W, Al-Jumeily D, Waraich A, Hussain A (2022). Gait Identification Using Limb Joint Movement and Deep Machine Learning. IEEE Access.

[4] Topham, L., Khan, W., Al-Jumeily, D. & Hussain, A. J. Human Body Pose Estimation for Gait Identification: A Comprehensive Survey of Datasets and Models. *ACM Comput. Surv*. (2022).

[5] Topham L, Khan W (2022). Liverpool John Moores University.

[6] Sepas-Moghaddam, A. & Etemad, A. *Deep Gait Recognition: A Survey*. 1–19 (2021). 10.1109/TPAMI.2022.3151865 35167443

[7] Wen J, Shen Y, Yang J (2022). Multi-View Gait Recognition Based on Generative Adversarial Network. Neural Process. Lett..

[8] MOTI. MOTI. *MOTI* moti.dk (2021).

[9] Varol G (2018). BodyNet: Volumetric inference of 3D human body shapes. European Conference on Computer Vision (ECCV).

[10] Chereshnev R, Kertész-Farkas A (2017). HuGaDB: Human gait database for activity recognition from wearable inertial sensor networks. International Conference on Analysis of Images, Social Networks and Texts.

[11] Chauhan, A. Gait Silhouette Dataset. *Kaggle* https://www.kaggle.com/watermasterz/gaitsilhouettedataset?select=GaitDatasetC-silh (2020).

[12] Verlekar TT, Soares LD, Correia PL (2018). Gait recognition in the wild using shadow silhouettes. Image Vis. Comput..

[13] Hebenstreit F (2015). Effect of walking speed on gait sub phase durations. Hum. Mov. Sci..

[14] Nunes JF, Moreira PM, Tavares JMRS (2019). GRIDDS - A Gait Recognition Image and Depth Dataset. In ECCOMAS Thematic Conference on Computational Vision and Medical Image Processing.

[15] Shutler J (2004). On a Large Sequence-Based Human Gait Database. Appl. Sci. Soft Comput..

[16] Hofmann M, Geiger J, Bachmann S, Schuller B, Rigoll G (2014). The TUM Gait from Audio, Image and Depth (GAID) database: Multimodal recognition of subjects and traits. J. Vis. Commun. Image Represent..

[17] Sheng, W. & Li, X. Multi-task learning for gait-based identity recognition and emotion recognition using attention enhanced temporal graph convolutional network. *Pattern Recognit*. **114** (2021).

[18] Office for National Statistics. Ethnicity and National Identity in England and Wales: 2011. https://www.ons.gov.uk/peoplepopulationandcommunity/culturalidentity/ethnicity/articles/ethnicityandnationalidentityinenglandandwales/2012-12-11 (2011).

[19] De Marsico, M. & Mecca, A. A survey on gait recognition via wearable sensors. *ACM Comput. Surv*. **52** (2019).

[20] Ahmad N, Ghazilla RAR, Khairi NM, Kasi V (2013). Reviews on Various Inertial Measurement Unit (IMU) Sensor Applications. Int. J. Signal Process. Syst..

[21] Palsson TS, Christensen SW, Thomsen MH, Hirata RP (2019). Assessment of range and quality of neck movement using a smartphone-based application. Musculoskelet. Sci. Pract..

[22] Rigoni M (2019). Assessment of shoulder range of motion using a wireless inertial motion capture device — A validation study. Sensors (Switzerland).

[23] Cao Z, Hidalgo G, Simon T, Wei SE, Sheikh Y (2021). OpenPose: Realtime Multi-Person 2D Pose Estimation Using Part Affinity Fields. IEEE Trans. Pattern Anal. Mach. Intell..

[24] Voight, J. *Quaternion algebras*. *Central Simple Algebras and Galois Cohomology*10.1007/978-3-030-56694-4 (Springer Nature, 2021).

