# Supplementary material for: A diverse and multi-modal gait dataset of indoor and outdoor walks acquired using multiple cameras and sensors
Source: Sci Data. 2023 May 26;10:320. doi: 10.1038/s41597-023-02161-8 (PMC10220063; doi:10.1038/s41597-023-02161-8)
Supplement: Supplementary file 1 — Supplementary Table 1. Overview of Existing Related Gait Datasets Including a Survey of the Sensors, Environments, and Diversity. [file 41597_2023_2161_MOESM1_ESM.docx]

Supplementary Table 1. Overview of Existing Related Gait Datasets Including a Survey of the Sensors, Environments, and Diversity.

| **Name** | **No. Subjects** | **No. Instances** | **Environment** | **Participant Diversity** | **Alternative Clothing / Appearance** | **Sensors** | **Labelling** | **Camera angles** | **Comment / Description** |
| --- | --- | --- | --- | --- | --- | --- | --- | --- | --- |
| **HuGaDB** | **18** | **679,073 frames** | **Indoor** | **Limited diversity (e.g., only 4 females particpated).** | **N/A** | **6 IMUs (upper legs, lower legs), feet, 2 EMGs.** | **Activities (e.g., walking, sitting).** | **N/A** | **Self-selected speeds. No video recordings.** |
| Gait Silhouette Dataset | **30** | **300 walks**  **100,000 frames** | **Unknown** | **Not described.** | **No.** | **Digital camera.** | **None.** | **1** | **Silhouette images of walking.**  **No original RGB.**  **No variation of angle.** |
| **IST gait database** | **21** | **252 walks** | **Outdoors** | **Not described.** | **No.** | **Digital Camera.** | **None.** | **4** | **In the wild shadows.** |
| **Human Gait Phase Dataset** | **21** | **25,306 steps** | **Indoors** | **Limited age, height, and mass diversity. Diversity in gender split.** | **No.** | **Motion capture.** | **3D motion tracking. Ground reaction forces.** | **1** | **Motion capture lab. Treadmill.**  **Only 615 steps manually labelled.**  **Not currently available online.** |
| **Gait Recognition Image and Depth Dataset (GRIDDS)** | **35** | **350 walks** | **Indoors** | **Limited.** | **Yes.** | **Depth camera, microphone.** | **Silhouette images.** | **1** | **3.6m range limit of sensor.**  **25 Labelled joints.** |
| **SOTON HiD** | **~100** | **~800 walks**  **~1,200 steps** | **Indoors.** | **Not described.** | **No.** | **Digital camera.** | **None.** | **4** | **Treadmill, lab-based, green screen.**  **Short walking sequences contain ~1.5 steps** |
| **TUM Gait from Audio, Image and Depth (TUM-GAID)** | **305** | **3,370 walks** | **Indoors.** | **Limited (particularly in age and height, ethnicity not recorded).** | **For 32 participants only. Limited appearance diversity due to weather conditions. With and without a backpack.** | **Depth camera, microphone.** | **None** | **2** | **Limited availability.**  **Shoe and backpack variation.** |
| **Ours** | **64** | **1,560 walks,**  **3,120 videos,**  **~10,920 steps (per angle),**  **748,000 frames,**  **Over 1 million sensor samples,**  **Over 56 million annotated keypoints** | **Indoors and outdoors with background variations.** | **Yes – gender, age, height, mass, and ethnicity.** | **Yes. Alternative clothing for each participant.** | **2 digital cameras, digital goniometer.** | **HPE (75 keypoints per image frame), anthropometry.** | **8 (45° increments)** | **Real-world environments. 8 viewing angles in 45° increments. Diverse participants. Alternative clothing for each participant.** |
