# Supplementary material for: A diverse and multi-modal gait dataset of indoor and outdoor walks acquired using multiple cameras and sensors
Source: Sci Data. 2023 May 26;10:320. doi: 10.1038/s41597-023-02161-8 (PMC10220063; doi:10.1038/s41597-023-02161-8)
Supplement: Supplementary file 2 — Supplementary Table 2. Anthropometry Information of the Participants. [file 41597_2023_2161_MOESM2_ESM.docx]

Supplementary Table 2. Anthropometry Information of the Participants.

| Participant No. | Gender | Age (years) | Height (cm) | Mass (kg) | Ethnicity |
| --- | --- | --- | --- | --- | --- |
| 1 | F | 27 | 153.0 | 60.0 | White British |
| 2 | F | 32 | 159.0 | 63.0 | White British |
| 3 | M | 30 | 175.0 | 65.0 | Asian |
| 4 | F | 57 | 153.0 | 95.0 | White British |
| 5 | M | 29 | 170.0 | 80.7 | White British |
| 6 | M | 27 | 177.0 | 108 | White British |
| 7 | F | 53 | 155.0 | 63.5 | White British |
| 8 | M | 27 | 169.0 | 80.0 | White British |
| 9 | F | 42 | 169.0 | 76.2 | White British |
| 10 | M | 40 | 178.0 | 77.0 | - |
| 11 | M | 37 | 180.0 | 70.0 | Asian |
| 12 | M | 37 | 171.0 | 67.0 | Mixed |
| 13 | F | 32 | 165.0 | 75.0 | British Mixed White -Asian |
| 14 | M | 21 | 185.0 | 95.0 | Asian |
| 15 | M | 19 | 181.0 | 90.0 | Asian |
| 16 | M | 32 | 180.0 | 86.0 | Asian |
| 17 | M | 55 | 172.7 | 90.0 | Asian |
| 18 | M | 21 | 175.3 | 85.0 | Asian |
| 19 | M | 30 | 182.9 | 75.0 | Asian |
| 20 | M | 28 | 183.0 | 70.0 | White British |
| 21 | M | 31 | 185.0 | 117.5 | White British |
| 22 | M | 25 | 183.0 | 79.5 | White British |
| 23 | M | 65 | 165.0 | 82.5 | White British |
| 24 | F | 42 | 152.0 | 133.0 | White British |
| 25 | M | 25 | 188.0 | 77.0 | White British |
| 26 | F | 24 | 162.5 | 57.0 | White British |
| 27 | M | 25 | 175.0 | 67.0 | African |
| 28 | M | 40 | 172.0 | 95.3 | Black British |
| 29 | M | 22 | 172.0 | 63.0 | White British |
| 30 | M | 20 | 173.0 | 74.0 | White British |
| 31 | F | 45 | 160.0 | 69.9 | White British |
| 32 | M | 44 | 167.0 | 79.0 | Black |
| 33 | M | 34 | 177.0 | 107.0 | Greek |
| 34 | F | 62 | 173.0 | 62.0 | White British |
| 35 | F | 50 | 165.0 | - | White British |
| 36 | M | 23 | 182.0 | 85.7 | White British |
| 37 | F | 52 | 162.5 | 52.0 | White British |
| 38 | M | 20 | 180.0 | 70.0 | Arab |
| 39 | M | 52 | 180.0 | 113.0 | White British |
| 40 | F | 34 | 165.0 | 51.0 | Asian |
| 41 | M | 47 | 190.5 | 88.9 | White British |
| 42 | - | - | - | - | - |
| 43 | M | 21 | 182.0 | 116.0 | Arab |
| 44 | F | 39 | 167.6 | 60.8 | White British |
| 45 | F | 40 | 137.2 | 133.4 | White British |
| 46 | M | 62 | 177.8 | 100.7 | White British |
| 47 | F | 29 | 170.2 | 57.2 | White British |
| 48 | F | 21 | 173.0 | 61.0 | White Dutch |
| 49 | F | 27 | 160.0 | 67.1 | White British |
| 50 | F | 31 | 157.5 | 83.5 | White Irish |
| 51 | F | 39 | 160.0 | 53.0 | White Italian |
| 52 | F | 26 | 152.4 | 74.9 | White British |
| 53 | F | 27 | 157.5 | 99.8 | White British |
| 54 | M | 47 | 177.0 | 72.0 | White Mixed |
| 55 | F | 23 | 157.0 | 56.0 | British Bangladeshi |
| 56 | F | 51 | 165.0 | 92.0 | White British |
| 57 | M | 61 | 174.0 | 98.5 | White British |
| 58 | F | 18 | 162.5 | 54.0 | White British |
| 59 | M | 36 | 180.3 | 85.0 | White British |
| 60 | F | 25 | 162.6 | 98.0 | White British |
| 61 | F | 26 | 170.2 | 80.0 | White British |
| 62 | M | 30 | 165.0 | 66.0 | Arab |
| 63 | M | 40 | 180.0 | 81.0 | African |
| 64 | M | 29 | 180.0 | 90.0 | White Polish |
| 65 | M | 26 | 192.0 | 110.0 | White Other |
| Summary | M: 37 **(57.81%)**  F: 27  **(42.19%)** | **35.14**  **(± 12.44)**  Max: 65  Min: 18 | **170.83**  **(± 10.86)**  Max: 192 Min: 137.16 | **81.51**  **(± 20.18)**  Max: 133.36  Min: 51 | White European: 62.5%  Black European,  Arab, Asian, African: 37.5% |
